# Extrapunitive and Intropunitive Individuals Activate Different Parts of the Prefrontal Cortex under an Ego-Blocking Frustration

**DOI:** 10.1371/journal.pone.0086036

**Published:** 2014-01-15

**Authors:** Takehiro Minamoto, Mariko Osaka, Ken Yaoi, Naoyuki Osaka

**Affiliations:** 1 Graduate School of Human Sciences, Osaka University, Suita, Osaka, Japan; 2 Graduate School of Letters, Kyoto University, Sakyo-ku, Kyoto, Japan; University of Medicine & Dentistry of NJ - New Jersey Medical School, United States of America

## Abstract

Different people make different responses when they face a frustrating situation: some punish others (extrapunitive), while others punish themselves (intropunitive). Few studies have investigated the neural structures that differentiate extrapunitive and intropunitive individuals. The present fMRI study explored these neural structures using two different frustrating situations: an ego-blocking situation which blocks a desire or goal, and a superego-blocking situation which blocks self-esteem. In the ego-blocking condition, the extrapunitive group (*n* = 9) showed greater activation in the bilateral ventrolateral prefrontal cortex, indicating that these individuals prefer emotional processing. On the other hand, the intropunitive group (*n* = 9) showed greater activation in the left dorsolateral prefrontal cortex, possibly reflecting an effortful control for anger reduction. Such patterns were not observed in the superego-blocking condition. These results indicate that the prefrontal cortex is the source of individual differences in aggression direction in the ego-blocking situation.

## Introduction

Anger often drives us to attack somebody who obstructs our goals. Psychologists have discussed how aggressive behaviors emerge for nearly one hundred years. According to the frustration-aggression hypothesis, anger, or hostile aggression, is evoked when an expected achievement of a desired goal is interfered [1]. Berkowitz [2] and Berkowitz and Harmon-Jones [3] have extended this idea by providing experimental evidences that physically unpleasant stimuli have enough potential to enhance the intensity of anger. Meanwhile, Anderson and Bushman [4] proposed the general aggression model, which posits how cognitive, affective, and arousal factors mediate personal and situational input to determine whether one should take impulsive or thoughtful aggressive actions.

A number of animal studies have investigated the neural structures that elicit and mediate these aggressive behaviors, including the neuroanatomical structures related to aggressive behaviors using lesion, electro-stimulating or pharmacological techniques [5]. Identified regions include the periaqueductal gray (PAG), hypothalamus, septal nuclei, amygdala, prefrontal cortex, bed nucleus of stria terminals (BNST), and nucleus accumbens. The PAG plays an important role in expressing defensive rage behavior, affecting several nuclei in the brain stem such as the locus coeruleus and central tegmental fields that are related to sympathetic arousal or hissing [5]. Subregions of the hypothalamus (e.g., the anterior hypothalamic area and ventromedial hypothalamus) were shown to affect aggressive behaviors by stimulating neurons located in the PAG [6]. The septal nuclei in the midbrain are also involved in aggressive behaviors, possibly by modulating neural excitations of the hypothalamus [7], while the central and medial nuclei of the amygdala appear to have different roles in aggression; the former functions to inhibit defensive rage, while the latter enhances it [8]. As for the prefrontal cortex (PFC), two regions are thought to be involved in processing aggression: the dorsolateral part and the orbitofrontal regions [9]. In monkeys, bilateral lesions in the dorsolateral PFC increased aggression, indicating that this region functions to inhibit reactive aggression [10], [11]. On the other hand, orbitally lesioned monkeys showed a decrease in aggression, suggesting that orbitofrontal regions are closely related to emotional processing [12], [13]. Furthermore, the lateral part of the prefrontal cortex appears to mediate reactive aggression by modulating neuronal activity of the medial hypothalamus via the mediodorsal thalamic nucleus [14], while the medial and lateral orbitofrontal cortex are known to interact with the medial nucleus of the amygdala and the medial hypothalamus, both of which mediate aggressive behavior [15]. Finally, the BNST, which receives inputs from the hypothalamus and amygdala, is associated with anger-related emotional processing [16], while the nucleus accumbens appears to affect aggression via neurochemical modulations of neurotransmitters such as dopamine and endogenous opioids [17].

Complementing the above neuroanatomical studies, neuroimaging studies have attempted to reveal the neural processes responsible for inducing aggressive behavior in humans. Some studies have localized the neural correlates of frustration, which can be a cause of aggressive behavior as proposed by Dollard et al. [1]. For example, a violation of reward expectation activates the ventrolateral prefrontal cortex (VLPFC) and the insula [18], while social frustration can also activate the VLPFC as well as the cingulate cortex [19]. Using the Taylor Aggression Paradigm, Kramer et al. [20] found that people have greater activation in the insula and anterior cingulate cortex when competing with an annoying frustrater who has given strong punishment. In terms of the general aggression model, these results suggest that stronger affective and arousal levels are reflected by neural activations that mediate aggressive behavior. Using the same paradigm, Lotze et al. [21] showed dissociative functions of the medial prefrontal cortex (mPFC) during an aggression-provoking situation. Specifically, the dorsal mPFC relates to cognitive operations that calculate the intensity of revenge, while the ventral mPFC is more involved in affective operations that observe the suffering caused to the opponent by the revenge.

The above studies mostly consider human aggression toward other people or objects. However, some people are more inclined to direct their aggression toward themselves, especially in social situations. For example, consider the situation in which someone makes an appointment with a person and visits him. Upon arrival, the other person says, “I have no time to see you today”. How does the appointment-maker respond? Some people would blame the appointee and say, “You should have told me you would be busy before I came here”. Others, however, blame themselves and say, “I should have contacted you one more time to confirm your availability today”. Such individual differences are common.

Saul Rosenzweig, an American psychologist, was the first researcher to classify apperceptive types of conscious reactions to frustration. He developed a picture frustration methodology [22] that classifies reactions to frustration into three categories: extrapunitive, intropunitive, and impunitive [23], [24]. *Extrapunitive* reactions are those in which one directs one's aggression toward the external environment; *intropunitive* reactions are those directed toward oneself; and *impunitive* reactions are those that condone the person who caused the frustrating situation. Of course, a healthy social life is adaptive and selects one of these three reaction styles accordingly. However, some individuals tend to select extrapunitive reactions or intropunitive ones more regularly, with little or no variation regardless of the incident. For example, clinical studies have reported that perpetrators of domestic violence show higher extrapunitive responses [25], and alcoholic males with depressive symptoms tend to exhibit intropunitive traits [26].

Although the existence of individual differences in reactions to frustration is obvious, few studies have investigated the neural structures that underlie these differences. Considering that individuals with extremely strong extrapunitive or intropunitive preferences often perform abnormal behaviors (e.g., abnormal aggression), it is critical to identify any brain structures closely linked to such traits. Therefore, the present study aimed to reveal the neural structures that differentiate extrapunitive and intropunitive individuals in a frustrating situation.

**Figure 1:**
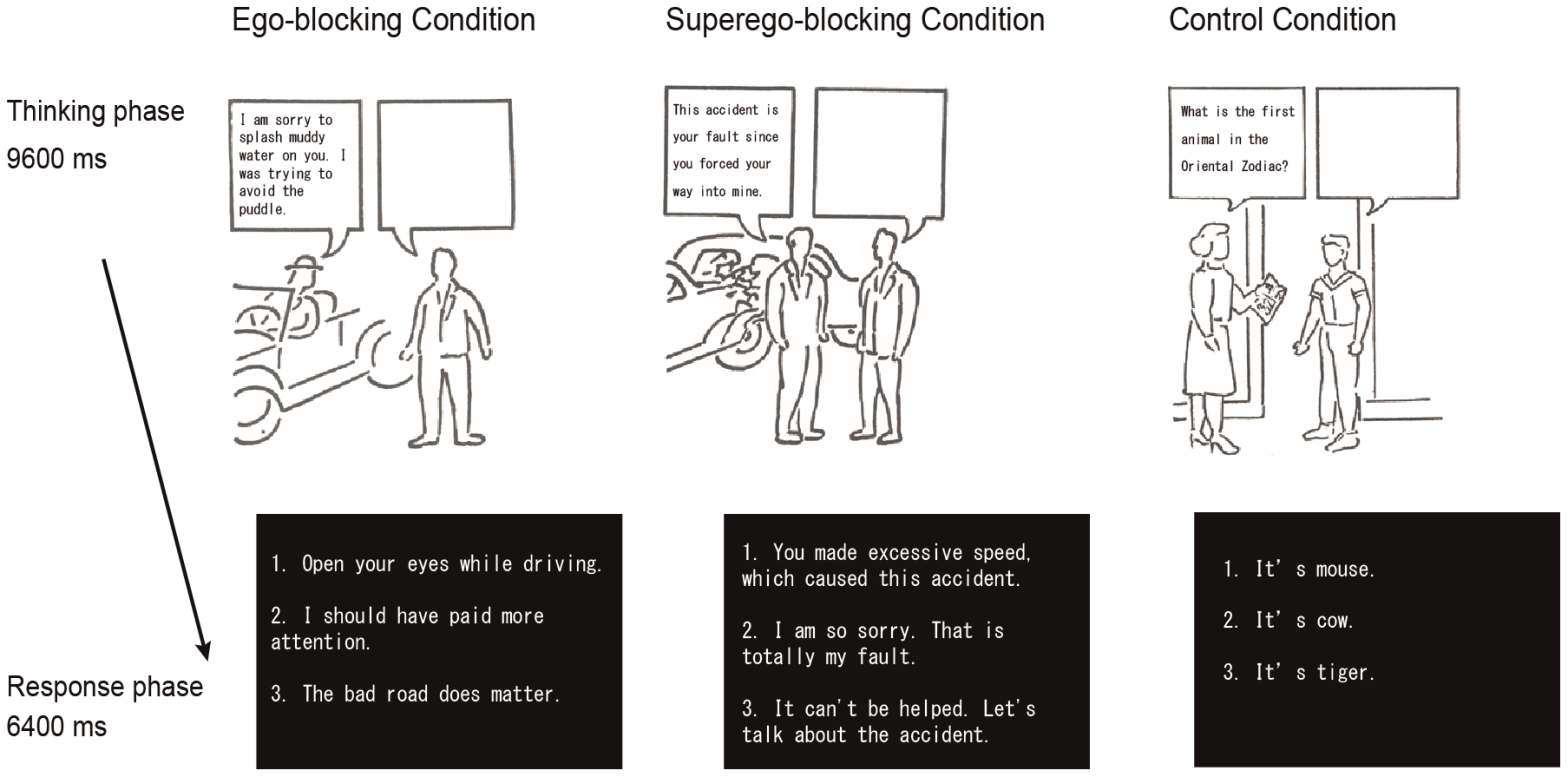
Schematic diagram of the experimental paradigm in the fMRI study. During a thinking phase, participants were instructed to think about how the right-hand person would reply in response to the remark by the left-hand person. In the answer phase, participants selected an answer from three candidates. Panels show a sample of the ego-blocking condition (left) and the superego-blocking condition (middle). In the control condition, participants were required to think about the answer to a question given by the right-hand person (right). Cartoon drawings in the figure were retrieved from the Manual for PF Study [30]. Permission to use was given by Sankyobo Ltd.

Using fMRI scans, we measured brain activity while participants experienced frustration induced by the picture frustration (PF) method. In this method, two characters face each other with speech balloons coming out of their mouths (see Fig. 1). Participants are instructed to think about how the right-hand character (frustratee) will respond to a statement from the left-hand character (frustrater), which causes frustration to the other, and to describe their answer in the frustratee's balloon. Two kinds of frustrating situations were prepared in the present study. One was the ego-blocking situation in which a frustrater disturbs or disappoints a frustratee, blocking the frustratee's desire (Fig. 1, left). The other was the superego-blocking situation in which a frustrater blames or reprimands a frustratee for an immoral behavior, blocking the frustratee's ideal self-image or self-esteem (Fig. 1, middle). Although both situations elicit frustration and lead to aggression, the ego-blocking situation can recruit automatic emotional processing, since a desire or specific goal is blocked for insignificant reasons. In this sense, the limbic area including the insula, the anterior cingulate cortex, and the amygdala might show stronger neural responses. On the other hand, in the superego-blocking situation, one tries to find a reasonable justification so that he or she can protect his or her self-esteem. Higher order cognitive operations might be recruited in this situation, activating the dorsolateral prefrontal cortex (DLPFC) and the posterior parietal cortex [27]. Using these different frustrating situations, we compared brain activity between extrapunitive and intropunitive groups.

The present study had two goals. The first was to dissociate neural structures involved with the ego-blocking situation from those involved with the superego-blocking situation. Because the two kinds of frustration can differ qualitatively (i.e., automatic emotional processing and cognitive operations, respectively), different brain regions may respond to each frustration. The second goal was to identify brain regions that differentiate extrapunitive and intropunitive individuals under frustrating situations. In light of the general aggression model, it is possible that differences in cognitive, affective, or arousal processing contribute to individual differences in aggression direction. Identifying such regions may help identify which factors (cognitive, affective, and arousal) are major contributors to individual differences in aggression direction.

We first compared the brain activation of the extrapunitive and intropunitive groups in each frustration condition. In addition, we localized those brain regions that activate differently between the two groups across the two frustrating conditions. Given that frustration is elicited by the other in the ego-blocking condition, while it is elicited by the self in the superego-blocking situation, we considered whether extrapunitive individuals may express more aggression toward the other in the ego-blocking condition, as they have a just cause to attack the frustrater. On the other hand, intropunitive individuals may experience stronger aggression toward themselves in the superego-blocking situation, as it is their own actions that have caused the frustrating situation. Therefore, we hypothesized that anger-related neural structures such as the ventral prefrontal cortex including the insula, the limbic regions or mid-brain structures have an interaction pattern of activation between the two experimental factors (frustration conditions and aggression direction).

## Materials and Methods

### Participants

Sixty-eight graduate and undergraduate students (mean age = 21.93, *SD* = 2.91, 27 females) participated in the pretest, with 35 agreeing to participate in the fMRI study (mean age = 23.05, *SD* = 2.53, 11 females). The pretest was included due to a characteristic of the Picture Frustration (PF) Study. Because this is a semi-projective test, where participants were allowed to give free answers to frustrater's comment, variety of answers was possible. As it was quite difficult to get their answers in the MR scanner but one needed participants' behavioral response to confirm that they performed the task, we decided to have obtained their answers prior to the fMRI experiment

Participants joined in the fMRI study reported normal or corrected to normal vision. Before the experiments, an experimenter gave a detailed description of the study to all participants, and each participant provided a written informed consent. The study protocol was approved by the Advanced Telecommunications Research Institute International prior to the experiments. In order to localize brain regions that differentiate intropunitive and extrapunitive groups, we performed group analysis, which compared individuals falling in the top quartile of extrapunitive score (*n* = 9) with those falling in the top quartile of intropunitive score (*n* = 9), using the extreme group design [28], [29]. This categorization was performed after the fMRI experiment, and solely based on the score of aggression direction, where other confounding variables were not controlled such as socioeconomic status or environmental exposure. Additionally, no rules of human categorization were required by any funding agencies. In order to examine a sampling bias in which attended participants could have stronger or weaker extrapunitive/intropunitive preference compared with non-attended participants, we compared extrapunitive and intropunitive scores between the attended (*n* = 35) and non-attended (*n* = 33) participants with a two-sample *t*-test. Scores of extrapunitive aggression did not differ between the two groups, *t* (66) = −1.14, *p* = .026, (*M* = 33.18 for the attended group, *M* = 37.03 for the non-attended group). Neither did scores of intropunitive aggression, *t* (66) = 0.51, *p* = .61, (*M* = 34.34 for the attended group, *M* = 33.31 for the non-attended group).

### Scoring of Aggression Type

At least 4 days prior to the fMRI experiments, participants took a Japanese version of the Rosenzweig picture frustration (PF) study for adults [30]. Two practitioners who majored in clinical psychology scored each sheet independently. Inter-rater reliabilities (Pearson's *r*) of the scores in each aggression direction are as follows: *r* (66) = .88 (*p*<.001) for extrapunitive and *r* (66) = .80 (*p*<.001) for intropunitive. Individual scores of each aggression direction were computed, averaging the scores reported by the two practitioners.

### Stimuli

In the fMRI study, a total of 18 pictures were selected from the Japanese version of the PF study. Those pictures were digitized using a scanner, and sentences in each speech balloon were retyped on image-editing software (Adobe Illustrator; Adobe Systems, Inc., San Jose, CA). One-third of the pictures were used for the ego-blocking condition (Situations 1, 4, 9, 13, 14, 18), another one-third for the super-ego blocking condition (Situations 5, 7, 10, 16, 17, 21), and the rest for the control condition. The control condition had six questions concerning general knowledge (e.g., “What is the first animal in the Oriental Zodiac”? Answer: Rat). An answer screen consisted of three choices, where one was the answer each participant described in the pretest and the other two were selected from samples of the PF study. This procedure was employed in order to confirm that participants read the sentences in the task and that their responses were consistent with those given in the pretest. In the control condition, an answer screen consisted of one target with two distractors. Stimuli were projected onto a screen through a mirror mounted on a head radiofrequency coil.

### Procedure

A trial began with the presentation of a cartoon drawing of interpersonal situations (Fig. 1). In the picture, two characters are depicted with speech balloons coming out of their mouths. Participants were instructed to silently read one or two sentences described in the speech balloons of the left-hand character and to think about how the right-hand person responded to the speech. Each cartoon was presented for 9600 ms. Following the cartoon picture, an answer screen was presented for 6400 ms, and participants were required to select one answer from three candidates by pressing a button on a response pad. In the ego-blocking and superego-blocking conditions, participants selected the answer which was closest to their thought. In the control condition (Fig. 1 right), they were instructed to select the correct answer. Each condition had six trials, and the order of the presentation was determined by Optsec2 (http://surfer.nmr.mgh.harvard.edu/optseq/). A rest period of 19200 ms was given between trials. Stimulus presentation and response retrieval were regulated with Presentation (Neurobehavioral Systems, Inc., Albany, CA).

The present study used an fMRI block design in which a relatively small number of blocks is sufficient for detecting brain activation related to a target psychological function, assuming that the brain activation associated with a specific psychological process (in this case frustration) is stable during the given block. This assumption was made even though behavioral indices supporting it were not collected. However, time-course data of the bilateral ventrolateral prefrontal cortex (VLPFC), which could reflect frustrating state [18], [19], showed sustained activation for 12.6 second (figure S1). Such duration of activation seems to meet a criterion of the block design, although it should be noted that the sustained activation might be correlated with a psychological processes other than frustration.

### fMRI Data Acquisition

Functional images were obtained using a 3.0-T MRI scanner (MAGNETOM Verio (3T), Siemens, Munich, Germany). Head motions were minimized with a forehead strap and comfortable padding around the participant's head. Functional images (223 images) sensitive to blood oxygen level-dependent (BOLD) contrasts were acquired by a single-shot echo-planar imaging sequence (TR = 3200 ms, TE = 30 ms, flip angle = 80°, 64×64 at 3 mm in-plane resolution, 3-mm thickness, 50 contiguous oblique axial slices parallel to the AC–PC line). The fMRI data will be available in the OpenfMRI (https://openfmri.org/). After the experimental scans, anatomical images were collected for all participants (TR = 2250 ms, TE = 3 ms, flip angle = 9°, voxel size = 1×1×1 mm).

### fMRI Data Analysis

Imaging data were analyzed with SPM5 (Wellcome Trust Center for Imaging, London, UK) running on Matlab 7.30 (Mathworks Inc., Sherborn, MA). Six initial dummy scans were discarded to eliminate any nonequilibrium effects of the magnetization; the remaining scans were included in the following analysis. Head motion was corrected, and coregistration of the functional and anatomical images was performed. Coregistered images were normalized onto a common brain space (the MNI template) and smoothed with a Gaussian filter (full width half maximum = 6 mm).

When modeling the functional images, we employed a high-pass filter (1/128 Hz) to cut off baseline drifts and an autoregression model (1) to correct the temporal correlated data. A total of seven regressors were convolved with the canonical HRF (Hemodynamic response function) and sustained boxcar function, including the thinking and response phases of the ego-blocking condition, the superego-blocking condition, and the control condition (9600 ms for the thinking phase; 6400 ms for the response phases). The last regressor was the resting period, whose duration was 19200 ms. Because our primary interest was brain activation during the thinking phase, we made a total of four contrast images for each participant, focusing on the thinking phase: ego>control, superego>control, ego>superego, and superego>ego. For the group-level analysis, we employed a random effect model. One-sample t-tests were performed in each contrast with a statistical threshold of *p*<.001 (uncorrected) and minimum of 10 contiguous voxels. The cluster threshold was applied to reduce type 1 error from multiple comparisons [31].

### Between Group Analysis

As described above, we employed extreme group analysis [28], [29], classifying participants falling in the top quartile of the extrapunitive scores as the extrapunitive group and those falling in the top quartile of the intropunitive scores as the intropunitive group. Mean extrapunitive scores in the extrapunitive and intropunitive groups were 52.5 (*SD* = 9.34) and 17.5 (*SD* = 5.03), respectively, and a two-sample t-test confirmed those means were significantly different (*t* (16) = 9.90, *p*<.001). Similarly, mean intropunitive scores in the extrapunitive and intropunitive groups were 24.44 (*SD* = 3.41) and 43.06 (*SD* = 4.49), respectively, and significantly different (*t* (16) = 9.90, *p*<.001).

In order to compare brain activations of the extrapunitive and intropunitive groups, we employed a two-sample *t*-test embedded in SPM5. First, we compared the brain activations of the two groups in each frustration condition. For this analysis, we created three contrast images per participant: main effects of the ego-blocking condition, the superego-blocking condition, and the control condition. Next, we examined brain regions that showed an interactive pattern of activation between the two factors. For this analysis we used two contrast images: ego-blocking – superego-blocking, and superego-blocking – ego-blocking. Interactions were tested by embedding these images in the two-sample *t*-test. In the group analysis, a statistical threshold of *p*<.001 (uncorrected) and minimum of 10 voxels was used. For illustration purposes, we created activation maps using a statistical threshold of *p*<.005 (uncorrected) and an extent threshold of 20 voxels.

### Statistical Analysis

Statistical analyses on the whole brain data were performed on the SPM 5. As for the behavioral data and percent signal change of ROIs, the STATISTICA software (StatSoft, Inc., Salsa. OK) was employed. Two-sample *t*-test was used in order to compare means of extrapunitive and intropunitive group. As for the interaction analysis, a two-way ANOVA (group×frustration condition) was employed. The Tukey's analysis was applied for post-hoc multiple comparisons. A statistical threshold of *p*<.05 was used as alpha-level.

## Results

### Behavioral Results

Participants mostly selected the same answers they had described in the pretest. The proportion selecting the same answers in the ego-blocking condition was 92.10% (*SD* = 13.51) and that in the superego-blocking condition was 92.19% (*SD* = 11.46). These percentages were not significantly different (*t* (34) = −.03, *p* = .97). In the control condition, most of the participants showed perfect performance (*M* = 97.14%, *SD* = 7.55).

### fMRI Results

We first examined differences in brain activation between the frustration conditions (the ego-blocking and superego-blocking conditions) and control condition (general knowledge condition), as this allowed us to evaluate whether the PF study activated the brain regions related to frustration. Next we investigated differences in brain activity between the ego-blocking and superego-blocking conditions, as these results indicate whether each frustration situation recruits different psychological processes. Finally, we compared the brain activity between the extrapunitive and intropunitive groups in the ego-blocking and superego-blocking conditions using a between-group result.

### Ego-blocking vs. Control and Superego-blocking vs. Control

**Figure 2:**
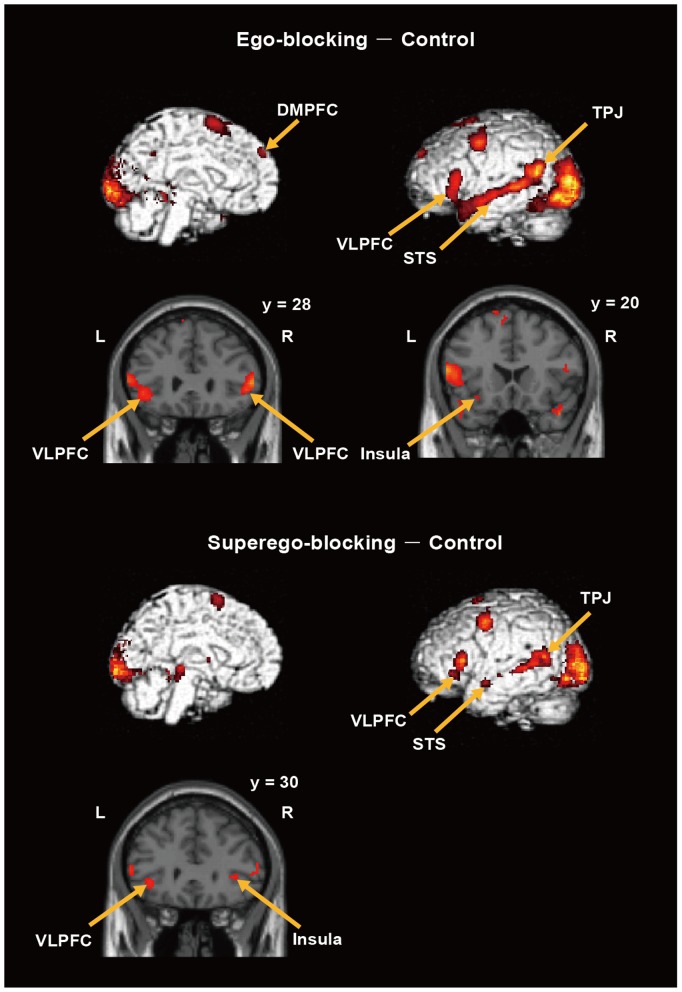
Brain regions showing greater activation in the ego-blocking condition than the control condition and in the superego-blocking condition than the control condition. DMPFC: Dorsomedial prefrontal cortex, VLPFC: Ventrolateral prefrontal cortex, TPJ: Temporoparietal junction, STS: Superior temporal sulcus. Brain images were templates embedded in SPM, whose use was permitted for a non-profit purpose.

As expected, the bilateral ventral prefrontal cortex corresponding to the inferior orbitofrontal cortices as well as the left insula showed greater activation in the ego-blocking condition than in the control condition (Fig. 2). Other notable regions showing greater activation in the ego-blocking condition include the dorsomedial prefrontal cortex, the bilateral temporal areas along with the superior temporal sulcus (STS) extending to the temporoparietal junction, and the precuneus (Fig. 2). Greater activation was also observed in the frontal and occipitotemporal areas. A summary of the results is shown in table 1, and beta weighted value of each region is provided in the figure S2.

**Table 1:** Brain regions showing greater activation in the ego-blocking condition than the control condition (*p*<.001 uncorrected, *cluster* >10 voxels).

Region	R/L	T-value	x	y	z	Voxels
Dorsomedial Prefrontal Cortex	L	5.49	−8	56	38	133
Dorsomedial Prefrontal Cortex	R	3.9	10	52	30	21
Ventrolateral Prefrontal Cortex	L	5.54	−46	26	−4	3567
Insula		4.26	−32	20	−12	
Superior Temporal Sulcus		8.21	−50	14	−30	
Superior Temporal Sulcus		8.12	−52	−8	−12	
Temporoparietal Junction		7.41	−48	−60	22	
Temporal Pole		5.33	−48	12	−26	
Ventrolateral Prefrontal Cortex	R	6.03	52	26	0	277
Insula		4.39	34	22	−16	
Supplementary Motor Area	L	6.7	−4	6	66	378
Precentral Gyrus	L	6.81	−46	−2	48	591
Precentral Gyrus	R	4.2	44	2	44	33
Superior Temporal Sulcus	R	5.63	56	−4	−14	728
Superior Temporal Sulcus		5.73	52	−32	2	
Temporal Pole		6.27	52	12	−18	
Temporoparietal Junction	R	5.41	62	−50	20	406
Precuneus	R	5.07	10	−56	30	219
Superior Occipital Gyrus	R	5.32	20	−68	38	211
Calcarine Fissure	L	11.87	−14	−94	−2	4706
Middle Occipital Gyrus		11.24	−24	−90	6	
Fusiform Gyrus		7.11	−30	−46	−8	
Lingual Gyrus	R	11.92	16	−90	−6	4137
Middle Occipital Gyrus		10.26	34	−88	10	
Calcarine Fissure		9.97	16	−92	4	
Fusiform Gyrus		9.94	30	−44	−8	

Similar to the ego-blocking condition, the ventral prefrontal cortex and the insula showed greater activation in the superego-blocking condition than in the control condition (Fig. 2). Greater activation was also found in the lateral temporal regions along the STS extending to the temporoparietal junction (Fig. 2) and in other regions including the frontal regions, the occipitotemporal areas, the hippocampus, the thalamus, and the mid-brain. The coordinates of these regions are summarized in table 2.

**Table 2:** Brain regions showing greater activation in the superego-blocking condition than the control condition (*p*<.001 uncorrected, *cluster* >10 voxels).

Region	R/L	T-value	x	y	z	Voxels
Ventrolateral Prefrontal Cortex	L	5.6	−50	22	8	653
		4.62	−38	28	−6	
Ventrolateral Prefrontal Cortex	R	5.04	58	26	8	195
Insula		4.41	36	30	2	
Supplementary Motor Area	L	5.09	−4	8	66	184
Precentral Gyrus	L	7.08	−42	2	50	524
Precentral Gyrus	R	3.96	42	−2	46	15
Temporal Pole	L	5.8	−52	−2	−14	199
Temporal Pole	R	5.98	52	8	−18	509
Superior Temporal Sulcus	R	5.77	52	−12	−10	
Superior Temporal Sulcus	L	5.25	−54	−32	0	1365
Temporoparietal Junction		5.99	−52	−58	16	
Temporoparietal Junction	R	3.57	66	−46	8	273
Calcarine Fissure	L	9.28	−14	−92	−6	2843
Middle Occipital Gyrus		7.82	−18	−100	2	
Fusiform Gyrus		6.07	−32	−44	−8	
Superior Occipital Gyrus	R	10.68	18	−98	8	2862
Fusiform Gyrus		8.74	34	−44	−8	
Lingual Gyrus		8.49	20	−88	−8	
Thalamus	L	4.73	−6	0	6	56
Hippocampus	L	4.44	−22	−30	−2	26
Mid Brain	L	6.99	−6	−30	−4	221
	R	4.82	6	−28	−2	

### Ego-blocking vs. Superego-blocking

**Figure 3:**
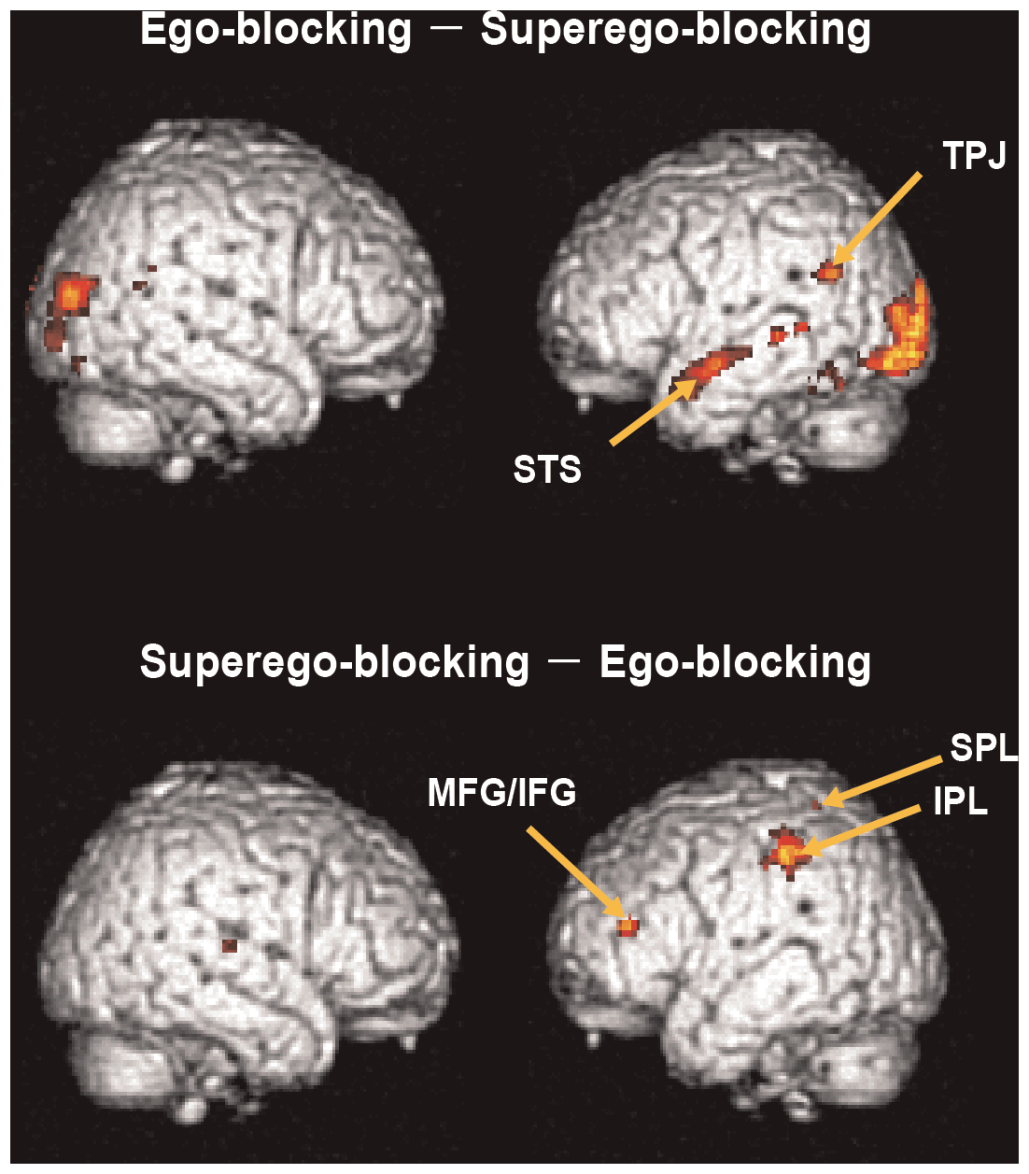
Activation contrast between the ego-blocking condition and the superego-blocking condition. TPJ: Temporoparietal junction, STS: Superior temporal sulcus, MFG/IFG: Middle/Inferior frontal gyrus, SPL: Superior parietal lobe, IPL: Inferior parietal lobule. Brain images were the templates embedded in the SPM, and its usage was permitted for a non-profit purpose.

Compared with the superego-blocking condition, the ego-blocking condition showed greater activation in the bilateral temporoparietal junctions, the right precuneus, the left temporal regions following the superior temporal sulcus, and the bilateral occipitotemporal regions (Fig. 3). In contrast, the superego-blocking condition showed greater activation in the left middle/inferior frontal gyrus, the left superior parietal cortex, the left inferior parietal cortex, the right posterior insula, the left putamen, and the right thalamus (Fig. 3). The coordinates of these regions are summarized in table 3.

**Table 3:** Comparison of brain activation in the ego-blocking condition and the superego-blocking condition (*p*<.001 uncorrected, *cluster* >10 voxels).

Region	R/L	T-value	x	y	z	Voxels
Ego-Blocking>Superego-blocking						
Superior Temporal Sulcus	L	5.35	−52	−8	−12	276
Temporoparietal Junction	L	5.17	−42	−58	24	126
Temporoparietal Junction	R	3.92	62	−56	16	11
Angular Gyrus	R	3.56	48	−48	24	12
Precuneus	R	4.21	14	−52	24	68
Posterior Cingulate Cortex		4.15	8	−46	26	
Fusiform Gyrus	L	5.84	−28	−40	−12	268
Fusiform Gyrus	R	5.34	30	−46	−8	67
Superior Occipital Gyrus	L	6.11	−14	−98	20	782
Middle Occipital Gyrus		5.45	−36	−90	6	
Calcarine Fissure		5.23	−12	−98	−2	
Middle Occipital Gyrus	R	5.95	36	−86	14	282
Inferior Occipital Gyrus		3.48	38	−86	0	
Calcarine Fissure	R	4.56	8	−56	12	63
Superego-blocking>Ego-blocking						
Middle/Inferior Frontal Gyrus	L	5.31	−38	36	16	68
Superior Parietal Cortex	L	3.65	−40	−48	64	21
Inferior Parietal Cortex	L	5.26	−54	−34	46	247
Insula	R	4.74	34	−22	12	41
Putamen	L	4.96	−34	−16	−4	74
Thalamus	R	4.77	16	−24	0	20

### Between Group Analysis

**Figure 4:**
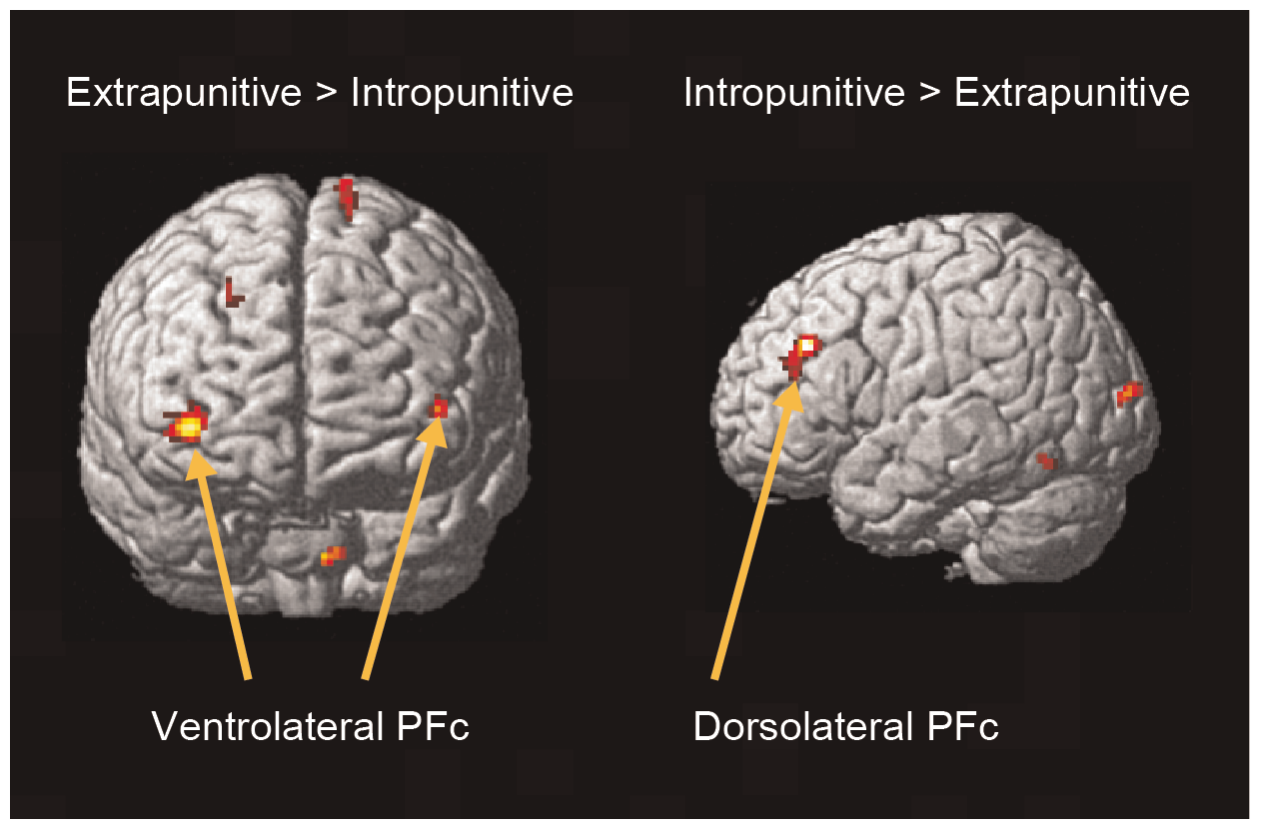
Comparisons of brain activation between extrapunitive and intropunitive individuals in the ego-blocking condition. Extrapunitive individuals showed greater activation in the bilateral ventrolateral prefrontal cortex than intropunitive individuals. On the other hand, intropunitive individuals showed greater activation in the left dorsolateral prefrontal cortex than extrapunitive individuals. Brain images were the templates embedded in the SPM, and its usage was permitted for a non-profit purpose.

Using an extreme group design, we classified participants falling in the top quartile of extrapunitive scores as the extrapunitive group (*n* = 9) and those falling in the top quartile of intropunitive scores as the intropunitive group (*n* = 9). In the ego-blocking condition, the extrapunitive group showed greater activation in the bilateral ventrolateral prefrontal cortex (which includes the inferior orbitofrontal gyrus and the inferior frontal gyri), the right superior frontal gyrus, and the left precentral gyrus (Fig. 4). On the other hand, the intropunitive group showed greater activation in the left middle frontal gyrus and the left middle occipital gyrus (Fig. 4). The coordinates of these regions are summarized in table 4. Power analysis was also performed in each prefrontal region, using the pwr package running on R (http://cran.r-project.org/web/packages/pwr/index.html). For the analysis, we created three ROI (the bilateral VLPFC and the left DLPFC), which were sphere-shaped with 4 mm radius and their center coordinates were located in the peak voxel. Percent signal change of each region was extracting with the MarsBar tool box [32], which is shown in the figure S3. The values of effect size and power are as follows: right VLPFC (*d* = 0.70, *power* = 0.29), left VLPFC (*d* = 0.93, *power* = 0.46), and the right DLPFC (*d* = 0.65, *power* = 0.25). As for the superego-blocking condition, significant differences were not observed between groups.

**Table 4:** Comparison of brain activation between extrapunitive and intropunitive individuals in the ego-blocking condition (*p*<.001 uncorrected, *cluster* >10 voxels).

Region	R/L	T-value	x	y	z	Voxels
Extrapunitive>Intropunitive						
Ventrolateral Prefrontal Cortex	R	5.74	36	44	0	40
Inferior Frontal Gyrus	L	5.88	−42	38	6	14
Superior Frontal Gyrus	R	4.38	20	28	40	13
Precentral Gyrus	L	4.49	−38	0	30	12
Intropunitive>Extrapunitive						

Region	R/L	T-value	x	y	z	Voxels
Middle Frontal Gyrus	L	4.5	−46	36	30	15
Middle Occipital Gyrus	L	5.06	−22	−94	12	24

**Figure 5:**
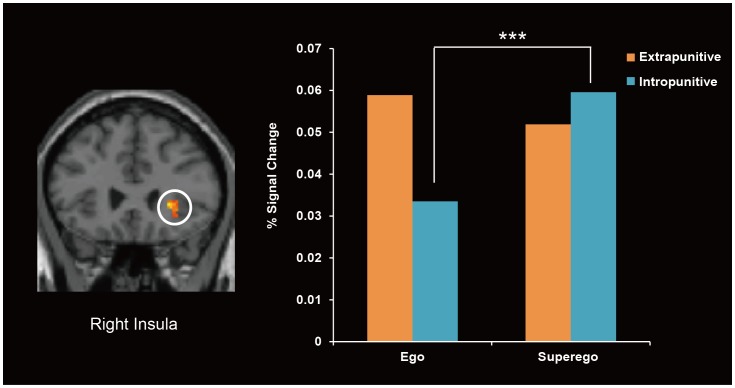
Brain regions showing interactive activation between the frustration condition and the punitive group. The intropunitive group showed greater activation in the superego-blocking condition than in the ego-blocking condition. No difference in activity was observed in the extrapunitive group.

An Interaction between the frustration conditions and aggression direction was found only in the right insula (Fig. 5). In order to examine the direction of the interaction, a complementary ROI analysis was performed. We extracted the percent signal change of the activated cluster and ran two-way mixed ANOVA for comparison. The results showed a significant interaction between two experimental factors (*F* (1, 16) = 53.51, *p*<.001), while Tukey's multiple comparisons test showed a significant difference in activation between the ego-blocking and superego-blocking condition in the intropunitive group (*p*<.001) (Fig. 5).

## Discussion

The present study addressed two questions. The first considered whether two qualitatively different frustrations, those provoked by ego-blocking situations and those provoked by superego-blocking situations, are processed through distinctive neural and psychological processes. The second considered which factors in the general aggression model contribute to individual differences in the aggression direction (i.e., extrapunitive or intropunitive). These questions were investigated from the perspective of neural activation.

Using fMRI, we measured brain activity while participants performed the picture frustration (PF) study. In both the ego-blocking and superego-blocking conditions, several brain regions showed greater activity compared with the control condition in which general knowledge was tested. These regions include the VLPFC, the insula, the STS, the temporoparietal junction, the temporal pole, the supplementary motor area, the precentral gyrus, and the occipital regions. Considering previous studies on frustration [18], [19], [20], activation of the VLPFC and the insula may reflect subjective experiences of frustration. At the same time, our conclusions should be taken with caution because the fMRI method localizes brain regions that correlate with experimental manipulations and the present study employed a task which required multiple cognitive and affective functions. In fact, the VLPFC is reported to be involved in decision-making [33] and the control of memory retrieval [34], while activation of the insula was shown to be associated with a wide range of emotions beyond anger [35]. Therefore, we cannot exclude the possibility that these other functional components of the VLPFC and insula contributed to our results. Nevertheless, the present study provides empirical data that links specific neural structures to frustration. One structure we did not observe to be activated was the anterior cingulate cortex (ACC), which disagrees with other studies [19], [20]. This result may be because participants did not experience social pain during our study.

In addition to the ventrolateral prefrontal cortex and the insula, greater activation was observed in temporal regions including the temporal pole, the STS, and the temporoparietal junctions in both the ego- and superego-blocking conditions. As our task required participants to think about the mental states of the frustrater, these activations may relate to mentalizing processes including cognitive processes such as the perception of the intentional behaviors of the cartoon characters (STS) and the retrieval of personal experiences to appreciate the frustratee's emotions (temporal pole). Previous studies with theory of mind tasks found activation of these same regions, supporting our interpretation [36], [37]. However, because these regions also carry multiple functions, such as language comprehension [21] and bottom-up attentional processing [20] in addition to social cognitive processes, special attention needs to be taken when interpreting the results.

Compared with the control condition, greater activation was found in the dorsomedial prefrontal cortex (DMPFC) in the ego-blocking condition, but not in the superego-blocking condition. Because a number of studies have shown the DMPFC functions to refer to another's emotional state [38], [39], we interpreted activation of the DMPFC in the ego-blocking condition as reflecting a mentalizing function. However, another study has shown that the DMPFC is involved in emotional judgment [40], which could contribute to the task performance in the present study, since participants were required to consider the frustratee's emotional state. On the other hand, DMPFC activation was not observed in the superego-blocking condition. One interpretation of this result may be that the superego-blocking condition requires an executive function rather than mentalizing to cope with the situation. In other words, participants might allocate their cognitive resources to resolve superego-blocking situations given that they projected themselves as a frustratee who caused a frustrating situation and was thus to be blamed or reprimanded by the frustrater. Consistent with this theory is that the left middle/inferior frontal gyri and the left superior/inferior parietal lobe, which both support working memory, show greater activation in the superego-blocking condition than the ego-blocking condition [41].

When directly compared with the superego-blocking condition, the following regions showed greater activation in the ego-blocking condition: the bilateral temporoparietal junctions, the left STS, and the medial parietal cortex including the precuneus and posterior cingulate cortex, all of which play an essential role for empathy and mentalizing [42], [43]. These activations may indicate that participants make more effort to attribute a frustratee' mental state to themselves and consequently feel more empathy in the ego-blocking condition than in the superego-blocking condition. Activation of the left STS is consistent with the requirement of a third-person perspective [44]. That study suggested the activation could reflect retrieval of episodic memory in order to build a representation of the lay's person knowledge. Therefore, the STS activation in the present study might be related to episodic memory of personal experiences that provide deeper understanding of the frustratee's mental state.

Contrary to our expectation, activation of the limbic areas did not differ between the ego-blocking and superego-blocking conditions. This null result can be attributed to the nature of the present task. As discussed above, the PF study is less likely to elicit social pain, which is associated with activation of the anterior cingulate cortex. Similarly, the PF study does not contain threatening stimuli such as a fearful face, which could activate the amygdala [45]. Therefore, the ego-blocking situation seems not to activate automatic emotional processing; rather it activates neural networks related to mentalizing and empathy.

The opposite contrast (superego-blocking>ego-blocking) showed greater activation of the lateral prefrontal cortex and the posterior parietal cortex, which correspond to a working memory network [41]. Interestingly, these regions have also been reported to be active when resolving moral dilemmas. For example, greater activation of the middle/inferior frontal gyri and the inferior parietal lobe has been seen when participants face an impersonal moral dilemma [46], [47], indicating these regions function to resolve a dilemma by driving the working memory system or other higher-order cognitive processing such as abstract reasoning or problem solving. The same process might operate during superego-blocking situations in the present study. In other words, participants could drive a higher-order cognitive system in order to resolve a frustrating situation that threatens their self-esteem. Moreover, the activation was specific to the left side of the fronto-parietal regions. According to previous studies, the left fronto-parietal network functions as the phonological loop of verbal working memory [48], which plays a significant role in problem solving [49]. Therefore, the activation of the left fronto-parietal regions could also reflect higher problem solving in order to overcome superego-blocking situations and protect self-esteem.

Differences were further seen between extrapunitive individuals and intropunitive individuals in the ego-blocking condition, as distinct parts of the prefrontal cortex were activated. Extrapunitive individuals showed greater activation of the ventrolateral prefrontal cortex, which corresponds to the lateral orbitofrontal cortex. Previous neuroimaging studies have reported that this region increases its activity in response to anger-related stimuli [50], [51]. Therefore, its greater activation here can be regarded as elevated anger, which can lead to an attack on the frustrater. On the other hand, the intropunitive group showed greater activation of the left middle frontal gyrus, which is typically referred to as the dorsolateral prefrontal cortex (DLPFC). Given that the DLPFC plays a critical role in cognitive control [52], intropunitive individuals can recruit this system to reduce the anger provoked in the ego-blocking situation. According to Wilkowski and Robinson [53], individuals with low levels of trait anger possess higher cognitive control, arguing that intropunitive individuals use more psychological processes. Considering the fact that anger suppression is positively correlated with depression and guilt [54], a reduction of anger might strengthen the self-blaming behavior of intropunitive individuals. As for the left-specific activation of the DLPFC, Abe et al. [55] proposed that the left region functions to falsify or inhibit truth responses in a deception paradigm. If so, intropunitive individuals might falsify their real aggressive feeling toward others. Therefore, taken together, in a situation where other people block a goal or desire, individuals with extrapunitive tendencies can activate an affective component while those with intropunitive tendencies drive a cognitive component of the general aggression model [4]. These components may act as core psychological mechanisms that differentiate extrapunitive and intropunitive individuals. These conclusions, however, would benefit from further experiments that examine a causal relationship between the dissociative parts of the prefrontal cortex and individual differences in aggression direction that use non-invasive brain stimulation approaches.

In the superego-blocking condition, on the other hand, group differences in brain activity were not found. This result may be because the frustratee instigated the situation. Both groups then aim to resolve a frustrating situation that potentially harms self-esteem in a manner that activates the fronto-parietal network.

One region that showed different patterns of activation between the extrapunitive and intropunitive groups across the two frustrating conditions was the right insula. While extrapunitive individuals showed equivalent activation across conditions, intropunitive individuals showed greater activation of this region in the superego-blocking conditions than in the ego-blocking condition. Assuming that activation of the right insula reflects the level of frustration, our observations may indicate that intropunitive individuals experience greater frustration in the superego-blocking condition, possibly due to higher self-blame.

Dopamine levels in the DLPFC may explain why different parts of the prefrontal cortex were activated between the extrapunitive and intropunitive groups in the ego-blocking condition but not in the super-ego blocking condition. Dopamine levels are increased in the DLPFC when monkeys perform a working memory task [56]. This dopamine elevation could somehow decrease GABA levels in the ventrolateral prefrontal cortex, which reduces aggressive behavior [15], and possibly enhances serotonin level in the periaqueductal gray through the medial hypothalamus. In the present study, dopamine in the left DLPFC could have increased in the ego-blocking condition in the intropunitive group to decrease GABA in the ventrolateral prefrontal cortex and thus reduce aggression. In contrast, the extrapunitive group may not have experience this increase in DLPFC dopamine, preventing the subsequent GABA and aggression effects. On the other hand, in the superego-blocking condition, where more working memory resources could be demanded, dopamine release might be increased in both the extrapunitive and intropunitive groups. Thus, GABA levels in the ventrolateral prefrontal cortex would be reduced similarly, eliminating any differences in prefrontal activation between the groups. Further neurochemical and neurophysiological studies are needed to verify this hypothesis.

Despite our conclusions, the present study has several methodological limitations. fMRI methods only test correlations between BOLD signals and experimental manipulations; causal relationships should be investigated with an interventional approach such as transcranial magnetic stimulation or transcranial direct current stimulation. It should be also noted that the sample sizes of the extrapunitive and intropunitive groups were small, weakening the statistical power. Another issue is the lack of a self-report measure to the task. Although several PF studies are thought to require executive function and entail empathy, where scores of the PF study was correlated with impulsive responses highly depending on the executive function, performance of problem-solving, and traits of empathy under the sleep-deprived condition [57], validity tests such as a measurement of the subjective experience are critical in order to assert whether the task requires the cognitive or emotional processes that are predicted.

In summary, the present study revealed that extrapunitive and intropunitive individuals employ different parts of the prefrontal cortex when facing an ego-blocking frustrating situation. Considering that individuals with strongly extrapunitive or intropunitive preferences can show abnormal behaviors, further study is expected to reveal the neurobiological basis of the individual differences for treatment of such behavioral problems.

## Supporting Information

Figure S1 **Time course data of the bilateral ventrolateral prefrontal cortex (VLPFC).** Activation of the regions reached to peak 6.4 second after the trial onset. Their activation was greater until the time-point of 19.2 second. Percent signal change was computed, subtracting BOLD signal at the trial onset (0 second) from each time point. The bilateral VLPFC ROIs were sphere shaped with 4 mm radius whose center coordinate was determined using the contrast of (Ego-blocking - Control). The error bar represents standard error of the mean.
(TIF)

Figure S2 **Beta weight value of each brain region activated in the ego-blocking condition in comparison to the control condition.** According to the figure, difference in activity between the ego-blocking condition and the control condition was due to increased activation in the ego-blocking condition, but not due to decreased activation in the control condition. All the regions showed similar pattern; bilateral medial prefrontal cortex (MPFC), ventrolateral prefrontal cortex (VLPFC), insula, temporoparietal junction (TPJ) and superior temporal sulcus (STS).
(TIF)

Figure S3 **Percent signal change of the bilateral ventrolateral prefrontal cortex (VLPFC) and the left dorsolateral prefrontal cortex (DLPFC) in extrapunitive and intropunitive group.** In the VLPFC, extrapunitive group showed greater activation in comparison to the intropunitive group, *t* (16) = 4.85, *p*<.001 (right VLPFC) and *t* (16) = 5.59, *p*<.001. On the other hand, in the left DLPFC, the intropunitive group showed greater activation, *t* (16) = −3.90, *p* = .0013. The error bar represents standard error of the mean.
(TIF)
