# Supplementary figures and images for: Extrapunitive and Intropunitive Individuals Activate Different Parts of the Prefrontal Cortex under an Ego-Blocking Frustration
Source: PLoS One. 2014 Jan 15;9(1):e86036. doi: 10.1371/journal.pone.0086036 (PMC3893263; doi:10.1371/journal.pone.0086036)

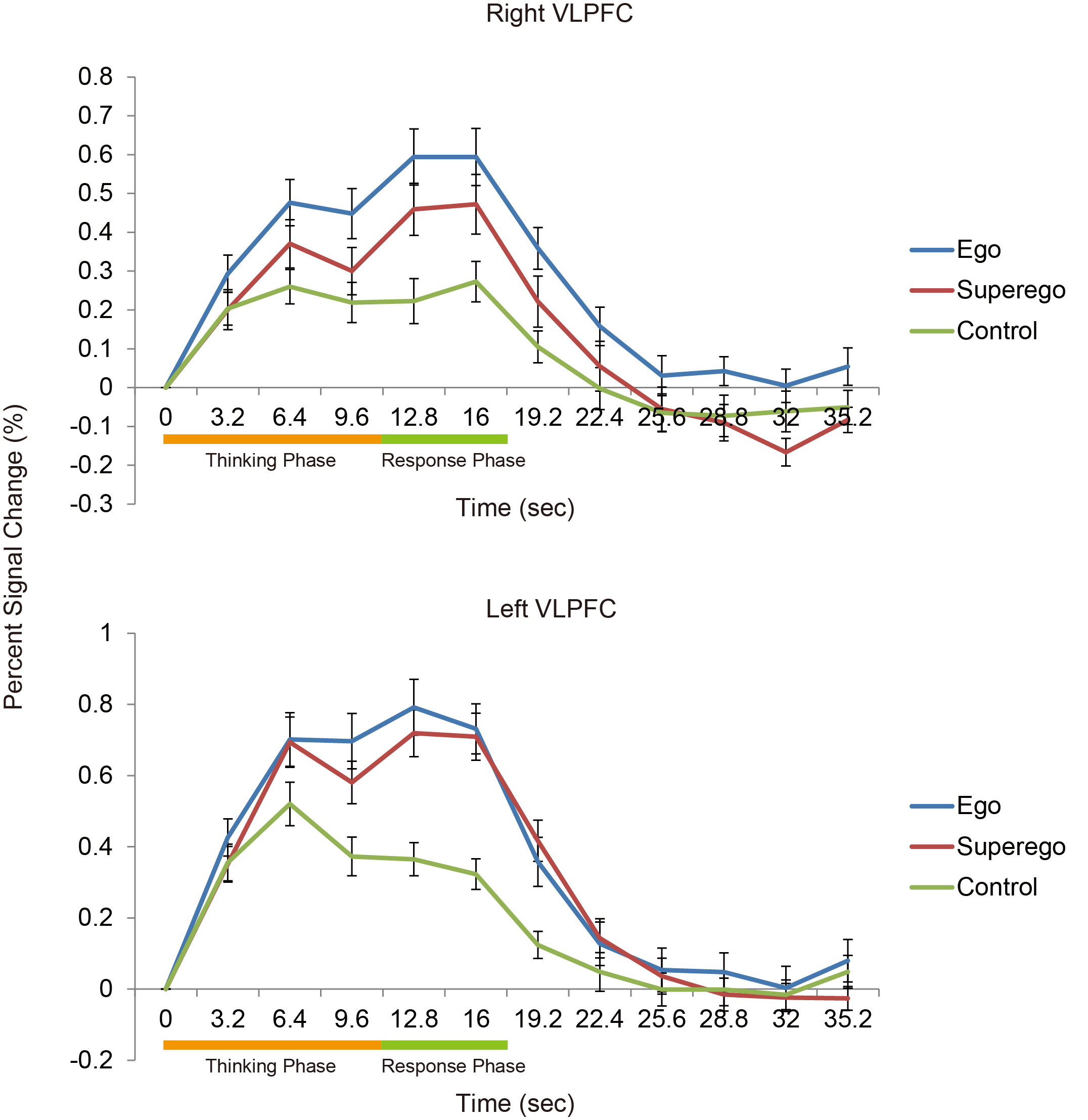

Supplement: Figure S1 — Time course data of the bilateral ventrolateral prefrontal cortex (VLPFC). Activation of the regions reached to peak 6.4 second after the trial onset. Their activation was greater until the time-point of 19.2 second. Percent signal change was computed, subtracting BOLD signal at the trial onset (0 second) from each time point. The bilateral VLPFC ROIs were sphere shaped with 4 mm radius whose center coordinate was determined using the contrast of (Ego-blocking - Control). The error bar represents standard error of the mean. (TIF) [file pone.0086036.s001.tif]

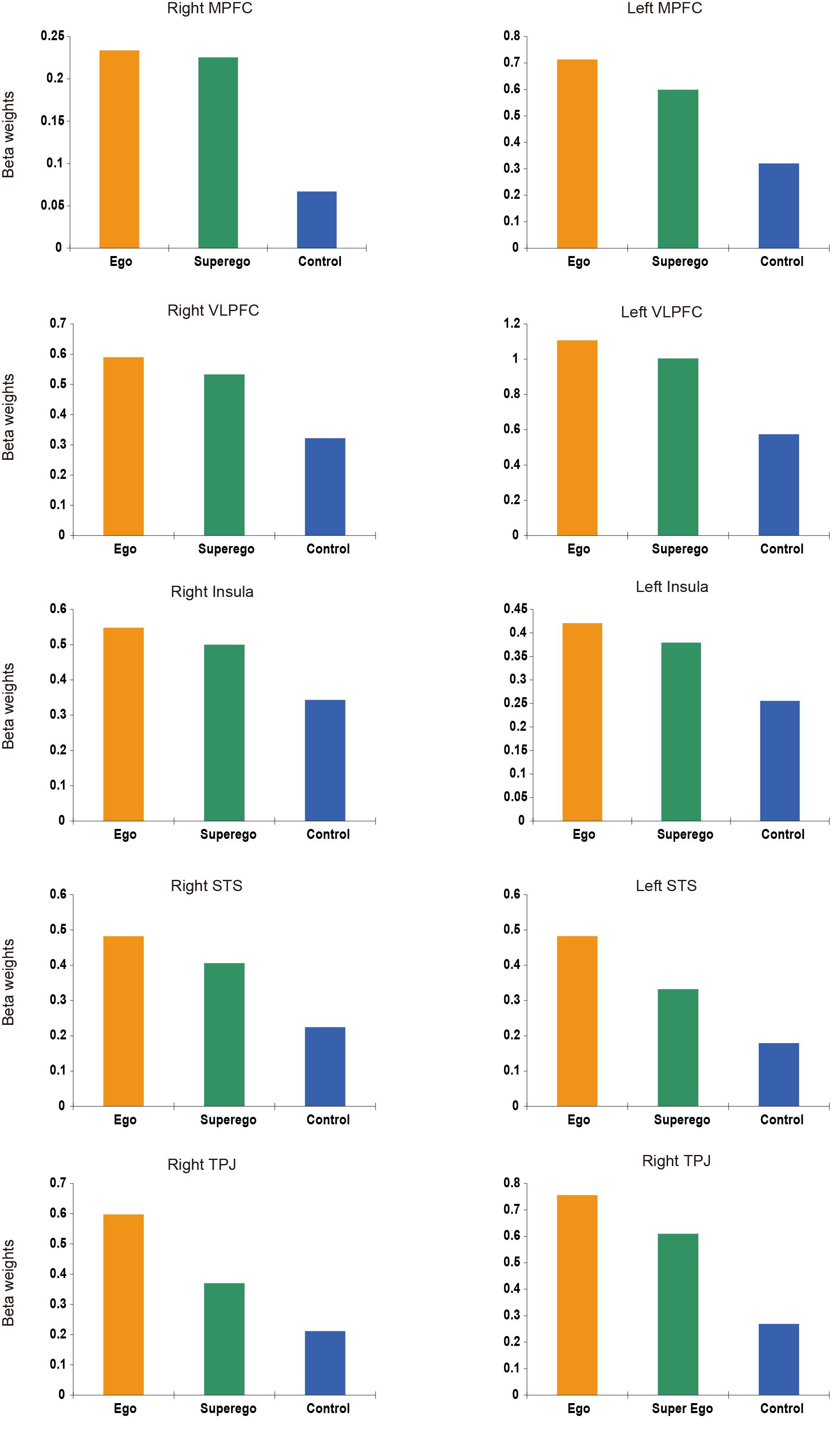

Supplement: Figure S2 — Beta weight value of each brain region activated in the ego-blocking condition in comparison to the control condition. According to the figure, difference in activity between the ego-blocking condition and the control condition was due to increased activation in the ego-blocking condition, but not due to decreased activation in the control condition. All the regions showed similar pattern; bilateral medial prefrontal cortex (MPFC), ventrolateral prefrontal cortex (VLPFC), insula, temporoparietal junction (TPJ) and superior temporal sulcus (STS). (TIF) [file pone.0086036.s002.tif]

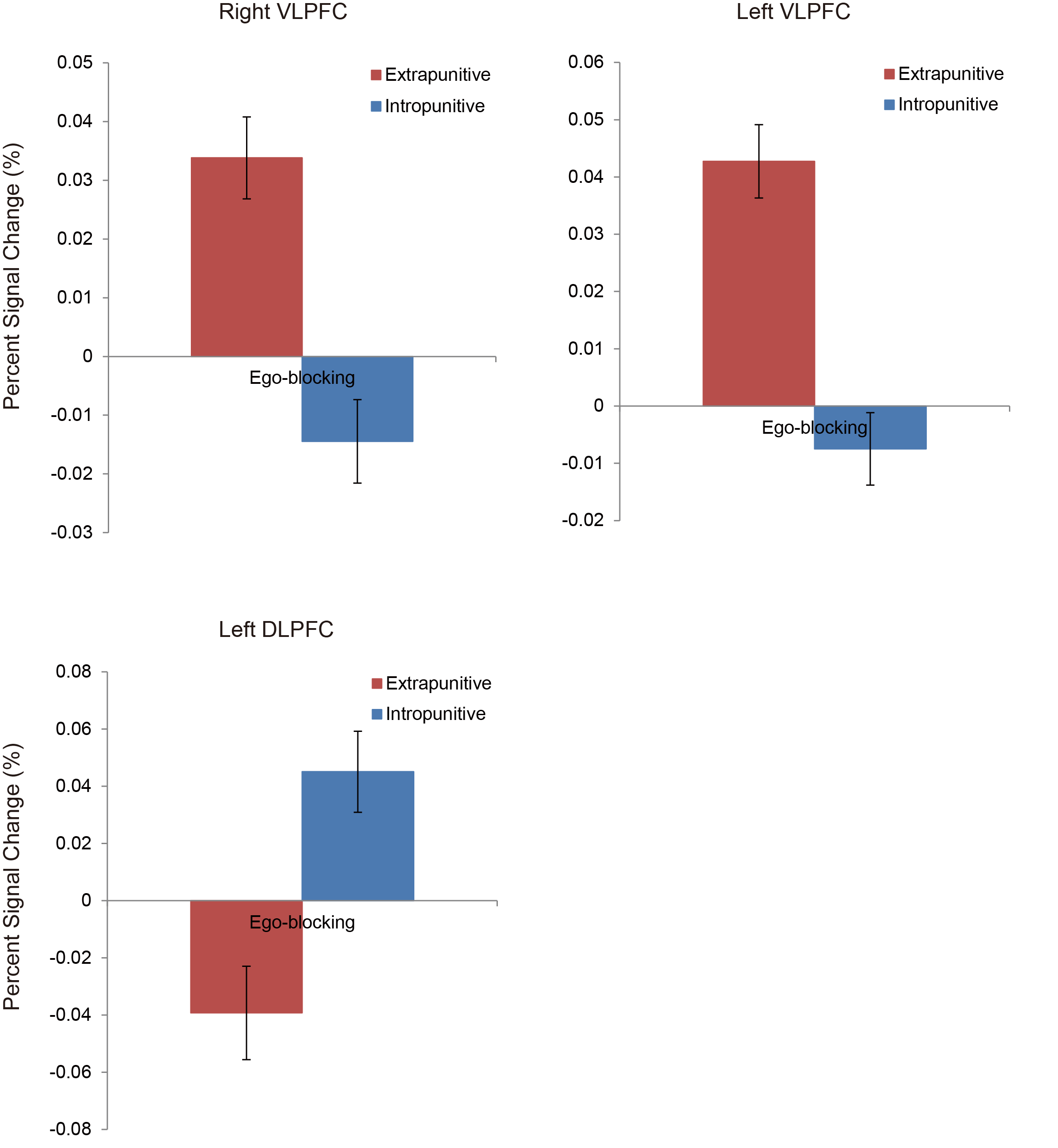

Supplement: Figure S3 — Percent signal change of the bilateral ventrolateral prefrontal cortex (VLPFC) and the left dorsolateral prefrontal cortex (DLPFC) in extrapunitive and intropunitive group. In the VLPFC, extrapunitive group showed greater activation in comparison to the intropunitive group, t (16) = 4.85, p<.001 (right VLPFC) and t (16) = 5.59, p<.001. On the other hand, in the left DLPFC, the intropunitive group showed greater activation, t (16) = −3.90, p = .0013. The error bar represents standard error of the mean. (TIF) [file pone.0086036.s003.tif]
